# 2,3,4,5,6-Penta­bromo­phenol

**DOI:** 10.1107/S1600536808028602

**Published:** 2008-09-13

**Authors:** Richard Betz, Peter Klüfers, Peter Mayer

**Affiliations:** a Department Chemie und Biochemie, Ludwig-Maximilians-Universität, Butenandtstrasse 5–13 (Haus D), 81377 München, Germany

## Abstract

The title compound, C_6_HBr_5_O, is the perbrominated derivative of phenol. The mol­ecule shows non-crystallographic mirror symmetry. Bond lengths between the C and Br atoms are normal. In the crystal structure, O—H⋯O hydrogen bonds connect the mol­ecules into infinite strands. Dispersive Br⋯Br contacts are observed. No significant π–π stacking is obvious.

## Related literature

For the structure of the perfluorinated derivative of phenol, see: Das *et al.* (2006 ▶); Gdaniec (2007 ▶). For the structure of 2,3,4,5,6-penta­chloro­phenol, see: Sakurai (1962 ▶).
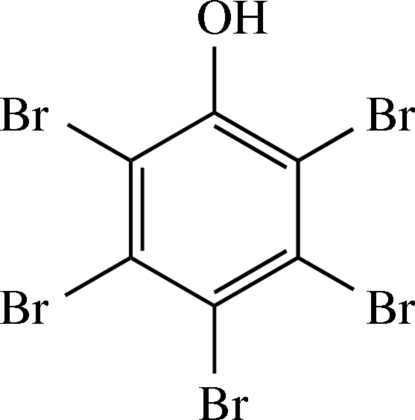


## Experimental

### 

#### Crystal data

C_6_HBr_5_O
*M* *_r_* = 488.57
Monoclinic, 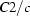

*a* = 32.3058 (15) Å
*b* = 3.9957 (2) Å
*c* = 16.1887 (8) Å
β = 112.118 (3)°
*V* = 1935.93 (17) Å^3^
*Z* = 8
Mo *K*α radiation
μ = 20.70 mm^−1^
*T* = 200 (2) K
0.28 × 0.08 × 0.05 mm

#### Data collection

Nonius Kappa CCD diffractometer
Absorption correction: multi-scan (*SADABS*; Sheldrick, 2001 ▶) *T*_min_ = 0.062, *T*_max_ = 0.355
13465 measured reflections
2219 independent reflections
1930 reflections with *I* > 2σ(*I*)
*R*_int_ = 0.054

#### Refinement

*R*[*F* ^2^ > 2σ(*F* ^2^)] = 0.030
*wR*(*F* ^2^) = 0.074
*S* = 1.03
2219 reflections
111 parameters
H-atom parameters constrained
Δρ_max_ = 0.88 e Å^−3^
Δρ_min_ = −1.02 e Å^−3^

### 

Data collection: *COLLECT* (Nonius, 2004 ▶); cell refinement: *SCALEPACK* (Otwinowski & Minor 1997 ▶); data reduction: *DENZO* (Otwinowski & Minor 1997 ▶) and *SCALEPACK*; program(s) used to solve structure: *SHELXS97* (Sheldrick, 2008 ▶); program(s) used to refine structure: *SHELXL97* (Sheldrick, 2008 ▶); molecular graphics: *ORTEP-3* (Farrugia, 1997 ▶) and *Mercury* (Macrae *et al.*, 2006 ▶); software used to prepare material for publication: *SHELXL97*.

## Supplementary Material

Crystal structure: contains datablocks I, global. DOI: 10.1107/S1600536808028602/rk2109sup1.cif

Structure factors: contains datablocks I. DOI: 10.1107/S1600536808028602/rk2109Isup2.hkl

Additional supplementary materials:  crystallographic information; 3D view; checkCIF report

## Figures and Tables

**Table 1:** Hydrogen-bond geometry (Å, °)

*D*—H⋯*A*	*D*—H	H⋯*A*	*D*⋯*A*	*D*—H⋯*A*
O1—H1⋯O1^i^	0.84	2.19	2.844 (4)	134

Symmetry code: (i) 
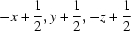
.

**Fig. 1.**
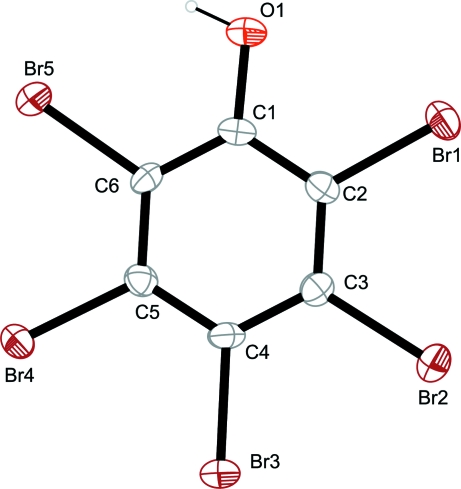
The molecular structure of the title compound, with atom labels. The displacement ellipsoids are drawn at 50% probability level. H atom is presented as a small sphere of arbitrary radius.

**Fig. 2.**
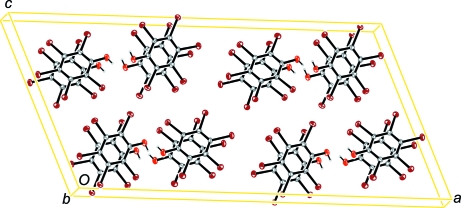
The crystal packing diagram, viewed along [010].

## supplementary crystallographic information

### Comment

During efforts to obtain tetraaryloxy derivatives of orthocarbonic acid it was interesting to determine the influence of bonding to one central carbon atom on geometric parameters of the ligands. Thus the crystal structure of 2,3,4,5,6-pentabromophenol was determined.

In the molecule (Fig. 1), C—C—C angles adopt values covering a range from 119.1 (3)° on the C atom bonded to the hydroxy group to 120.7 (3)° on one of the C atoms in *ortho*-position to the hydroxy group. The alterations between the C—C—C angles thus are less pronounced than in the perfluorinated derivative of phenol, where the angle on the C atom bearing the hydroxy group was found at a value slightly above 116° (Gdaniec, 2007). The values more closely resemble the ones apparent in the molecular structure of the perchlorinated derivative, yet the smallest C—C—C angle is not present on the C atom bearing the hydroxy group in that compound (Sakurai, 1962).

In the crystal structure H-bonds connect the molecules to infinite strands along [010] (Fig. 2). A bifurcation of the hydrogen bond between oxygen and one of the halogen atoms in *ortho*-position was not observed. This is in contrast to 2,3,4,5,6–pentachlorophenol, where the presence of such a bifurcated hydrogen bond was substantiated upon nuclear quadrupole resonance spectra for the Cl atoms (Sakurai, 1962). Additionally, dispersive Br···Br interactions between the Br atoms in both *meta*-positions to the hydroxy group are observed. The range of these interactions falls by about 0.1 Å below the sum of van der Waals radii of the respective atoms. These connect the molecules to chains along [001]. No significant π-stacking is apparent in the crystal structure. The molecular packing is shown in Fig. 3.

### Experimental

The compound was obtained commercially from Aldrich. Crystals suitable for X-ray diffraction were obtained upon recrystallization of the compound from boiling toluene.

### Refinement

The H atom was located in a difference map and refined as riding on its parent O atom with an *U*_iso_(H) = 1.5*U*_eq_(O).

### Crystal data

**Table:**

C_6_HBr_5_O	*F*(000) = 1760
*M**_r_* = 488.57	*D*_x_ = 3.353 Mg m^−^^3^
Monoclinic, *C*2/*c*	Mo *K*α radiation, λ = 0.71073 Å
Hall symbol: -C 2yc	Cell parameters from 8265 reflections
*a* = 32.3058 (15) Å	θ = 3.1–27.5°
*b* = 3.9957 (2) Å	µ = 20.70 mm^−^^1^
*c* = 16.1887 (8) Å	*T* = 200 K
β = 112.118 (3)°	Rod, colourless
*V* = 1935.93 (17) Å^3^	0.28 × 0.08 × 0.05 mm
*Z* = 8	

### Data collection

**Table:**

Nonius Kappa CCD diffractometer	2219 independent reflections
Radiation source: Rotating anode	1930 reflections with *I* > 2σ(*I*)
MONTEL, graded multilayered X-ray optics	*R*_int_ = 0.054
Rotation images; thick slices scans	θ_max_ = 27.6°, θ_min_ = 3.5°
Absorption correction: multi-scan (*SADABS*; Sheldrick, 2001)	*h* = −41→41
*T*_min_ = 0.062, *T*_max_ = 0.355	*k* = −4→5
13465 measured reflections	*l* = −21→21

### Refinement

**Table:**

Refinement on *F*^2^	Secondary atom site location: difference Fourier map
Least-squares matrix: Full	Hydrogen site location: inferred from neighbouring sites
*R*[*F*^2^ > 2σ(*F*^2^)] = 0.030	H-atom parameters constrained
*wR*(*F*^2^) = 0.075	*w* = 1/[σ^2^(*F*_o_^2^) + (0.0374*P*)^2^ + 5.8817*P*] where *P* = (*F*_o_^2^ + 2*F*_c_^2^)/3
*S* = 1.03	(Δ/σ)_max_ = 0.001
2219 reflections	Δρ_max_ = 0.88 e Å^−^^3^
111 parameters	Δρ_min_ = −1.02 e Å^−^^3^
0 restraints	Extinction correction: *SHELXL97* (Sheldrick, 2008)
Primary atom site location: structure-invariant direct methods	Extinction coefficient: 0.00087 (8)

### Special details

**Table:**

Geometry. All s.u.'s (except the s.u. in the dihedral angle between two l.s. planes) are estimated using the full covariance matrix. The cell s.u.'s are taken into account individually in the estimation of s.u.'s in distances, angles and torsion angles; correlations between s.u.'s in cell parameters are only used when they are defined by crystal symmetry. An approximate (isotropic) treatment of cell s.u.'s is used for estimating s.u.'s involving l.s. planes.
Refinement. Refinement of F^2^ against ALL reflections. The weighted R-factor wR and goodness of fit S are based on F^2^, conventional R-factors R are based on F, with F set to zero for negative F^2^. The threshold expression of F^2^ > 2σ(F^2^) is used only for calculating R-factors(gt) etc. and is not relevant to the choice of reflections for refinement. R-factors based on F^2^ are statistically about twice as large as those based on F, and R-factors based on ALL data will be even larger.

### Fractional atomic coordinates and isotropic or equivalent isotropic displacement parameters (Å^2^)

**Table:**

	*x*	*y*	*z*	*U*_iso_*/*U*_eq_	
Br1	0.204848 (13)	0.51248 (10)	0.41077 (2)	0.03029 (14)	
Br2	0.096769 (13)	0.38703 (10)	0.36125 (2)	0.02821 (13)	
Br3	0.024427 (13)	0.61492 (11)	0.16493 (3)	0.03106 (14)	
Br4	0.060474 (12)	0.97946 (11)	0.02144 (2)	0.02709 (13)	
Br5	0.169251 (13)	1.09979 (10)	0.07733 (2)	0.02651 (13)	
O1	0.22213 (8)	0.8423 (7)	0.26447 (18)	0.0289 (6)	
H1	0.2271	0.9643	0.2270	0.043*	
C1	0.17732 (11)	0.7978 (9)	0.2394 (2)	0.0213 (7)	
C2	0.16196 (12)	0.6421 (9)	0.2996 (2)	0.0216 (7)	
C3	0.11662 (12)	0.5889 (9)	0.2773 (2)	0.0211 (7)	
C4	0.08601 (11)	0.6883 (9)	0.1945 (2)	0.0213 (7)	
C5	0.10107 (12)	0.8416 (8)	0.1339 (2)	0.0209 (7)	
C6	0.14663 (12)	0.8957 (8)	0.1567 (2)	0.0197 (7)	

### Atomic displacement parameters (Å^2^)

**Table:**

	*U*^11^	*U*^22^	*U*^33^	*U*^12^	*U*^13^	*U*^23^
Br1	0.0248 (2)	0.0387 (3)	0.0229 (2)	0.00504 (16)	0.00385 (16)	0.00541 (16)
Br2	0.0300 (2)	0.0331 (2)	0.0244 (2)	−0.00243 (15)	0.01352 (16)	0.00349 (14)
Br3	0.0171 (2)	0.0443 (3)	0.0318 (2)	−0.00324 (15)	0.00924 (16)	0.00412 (16)
Br4	0.0201 (2)	0.0377 (2)	0.0212 (2)	0.00309 (15)	0.00524 (15)	0.00423 (14)
Br5	0.0238 (2)	0.0331 (2)	0.0255 (2)	−0.00301 (14)	0.01252 (16)	0.00230 (14)
O1	0.0160 (12)	0.0393 (16)	0.0315 (14)	0.0005 (11)	0.0091 (11)	0.0025 (12)
C1	0.0146 (16)	0.0222 (16)	0.0260 (17)	−0.0013 (14)	0.0066 (13)	−0.0031 (14)
C2	0.0188 (18)	0.0236 (18)	0.0201 (16)	0.0003 (14)	0.0048 (13)	−0.0015 (13)
C3	0.0237 (19)	0.0211 (16)	0.0208 (17)	−0.0001 (13)	0.0110 (14)	−0.0028 (13)
C4	0.0146 (16)	0.0261 (17)	0.0244 (17)	−0.0029 (14)	0.0087 (13)	−0.0038 (14)
C5	0.0196 (17)	0.0237 (17)	0.0184 (15)	−0.0001 (14)	0.0061 (13)	−0.0027 (13)
C6	0.0213 (17)	0.0215 (17)	0.0207 (16)	−0.0026 (14)	0.0129 (13)	−0.0009 (13)

### Geometric parameters (Å, °)

**Table:**

Br1—C2	1.884 (3)	C1—C6	1.389 (5)
Br2—C3	1.888 (4)	C1—C2	1.395 (5)
Br3—C4	1.886 (3)	C2—C3	1.387 (5)
Br4—C5	1.882 (3)	C3—C4	1.391 (5)
Br5—C6	1.886 (4)	C4—C5	1.390 (5)
O1—C1	1.360 (4)	C5—C6	1.393 (5)
O1—H1	0.8400		
			
C1—O1—H1	109.5	C5—C4—C3	119.7 (3)
O1—C1—C6	122.9 (3)	C5—C4—Br3	120.4 (2)
O1—C1—C2	117.9 (3)	C3—C4—Br3	120.0 (3)
C6—C1—C2	119.1 (3)	C4—C5—C6	119.8 (3)
C3—C2—C1	120.3 (3)	C4—C5—Br4	120.7 (3)
C3—C2—Br1	122.1 (3)	C6—C5—Br4	119.5 (3)
C1—C2—Br1	117.6 (3)	C1—C6—C5	120.7 (3)
C2—C3—C4	120.4 (3)	C1—C6—Br5	117.4 (3)
C2—C3—Br2	119.3 (3)	C5—C6—Br5	121.9 (3)
C4—C3—Br2	120.3 (3)		
			
O1—C1—C2—C3	−179.7 (3)	C3—C4—C5—C6	−0.2 (5)
C6—C1—C2—C3	−0.6 (5)	Br3—C4—C5—C6	179.7 (3)
O1—C1—C2—Br1	0.7 (4)	C3—C4—C5—Br4	−179.9 (3)
C6—C1—C2—Br1	179.8 (3)	Br3—C4—C5—Br4	0.1 (4)
C1—C2—C3—C4	0.4 (5)	O1—C1—C6—C5	179.4 (3)
Br1—C2—C3—C4	180.0 (3)	C2—C1—C6—C5	0.4 (5)
C1—C2—C3—Br2	−178.5 (3)	O1—C1—C6—Br5	−0.2 (5)
Br1—C2—C3—Br2	1.1 (4)	C2—C1—C6—Br5	−179.2 (3)
C2—C3—C4—C5	0.0 (5)	C4—C5—C6—C1	0.0 (5)
Br2—C3—C4—C5	178.9 (3)	Br4—C5—C6—C1	179.7 (3)
C2—C3—C4—Br3	−179.9 (3)	C4—C5—C6—Br5	179.6 (3)
Br2—C3—C4—Br3	−1.1 (4)	Br4—C5—C6—Br5	−0.7 (4)

### Hydrogen-bond geometry (Å, °)

**Table:**

*D*—H···*A*	*D*—H	H···*A*	*D*···*A*	*D*—H···*A*
O1—H1···O1^i^	0.84	2.19	2.844 (4)	134

Symmetry codes: (i) −*x*+1/2, *y*+1/2, −*z*+1/2.
